# Effect of a nutrition education intervention on sports nutrition knowledge, dietary intake, and body composition in female athletes: a randomized controlled trial

**DOI:** 10.3389/fnut.2026.1821345

**Published:** 2026-05-08

**Authors:** Macarena Veloso-Pulgar, Andreu Farran-Codina

**Affiliations:** 1 Departament de Nutrició, Ciències dels Aliments i Gastronomia, Facultat de Farmàcia i Ciències de l’Alimentació, Universitat de Barcelona, Barcelona, Spain; 2 Institut de Recerca en Nutrició i Seguretat Alimentària (INSA-UB), Universitat de Barcelona, Barcelona, Spain

**Keywords:** body composition, dietary intake, female athletes, nutrition education, nutritional knowledge

## Abstract

**Background/objectives:**

Adolescent female athletes often present insufficient dietary intake and limited sports nutrition knowledge (SNK). This study aimed to evaluate the effects of a short-term nutrition education intervention on SNK, dietary intake, and body composition in adolescent female handball players.

**Methods:**

A community-based randomized controlled trial was conducted. Four handball clubs were randomly assigned to either an intervention group (IG) or a control group (CG). The IG received a nutrition education program consisting of three 30-min face-to-face group sessions delivered by a registered dietitian. The CG did not receive any nutrition education intervention during the study period. SNK, dietary intake, Mediterranean diet adherence (MD), and anthropometric and body composition measurements were assessed at baseline and follow-up in both groups.

**Results:**

A total of 97 participants were included (IG = 60, CG = 37), aged 12 to 19 years (mean 15.8 ± 1.2 years). SNK increased significantly in the IG immediately after the intervention and at three-month follow-up compared with baseline (*p* < 0.05, effect size > 0.8). Baseline dietary records revealed, in both groups, insufficient energy intake (30 kcal/kg/day) and carbohydrate intake (2.7 g/kg/day), alongside a high total fat intake (1.5 g/kg/day). At baseline, 77.8% of the IG and 78.0% of the CG required improvements in adherence to the MD. No significant changes were observed in dietary intake, MD adherence, or body composition in either the IG or CG following the intervention.

**Conclusion:**

A brief nutrition education intervention effectively improved SNK in adolescent female athletes. However, these improvements did not translate into changes in dietary intake or body composition. These findings suggest that interventions incorporating behavioral strategies may be necessary to achieve significant dietary changes in this population.

**Clinical trial registration:**

ClinicalTrials.gov, identifier (NCT07460102).

## 1 Introduction

Adolescence is a critical stage of the human life course, characterized by marked physical, physiological, psychological, and social changes and increased energy and nutritional requirements essential for optimal growth and development (1). However, dietary habits during this period are often unstable and shaped by family, social, and cultural influences, with the progressive acquisition of dietary autonomy frequently associated with suboptimal eating patterns with a high intake of energy-dense, nutrient-poor foods (2). In adolescent athletes, these challenges are amplified by the additional demands imposed by training and competition, making adequate energy intake, balanced macronutrient distribution, and sufficient micronutrient supply fundamental for maintaining health and optimizing athletic performance (3, 4).

In female athletes, nutritional management is further complicated by sex-specific physiological factors such as hormonal fluctuations and the menstrual cycle, which may increase vulnerability to health and performance impairments. Prolonged insufficient energy intake can lead to low energy availability (LEA) and progress to relative energy deficiency in sport (REDs), a condition affecting multiple physiological systems, including bone health and reproductive function (5). Despite established nutritional guidelines for athletes (6), a substantial proportion of female athletes fail to meet these recommendations, commonly exhibiting inadequate carbohydrate intake, excessive fat consumption, and insufficient intake of key nutrients such as calcium, iron, and vitamin D (7–9).

Although dietary inadequacy is multifactorial, inadequate sports nutrition knowledge (SNK) represents a major contributing factor (10, 11). Nutritional literacy is essential for informed dietary decision-making, and higher levels of nutrition knowledge have been associated with healthier eating behaviors and enhanced performance across competitive levels (8, 12). Nevertheless, consistently low SNK levels have been reported among female athletes from different sports disciplines (8, 12–17). In response, nutrition education interventions, delivered through both face-to-face and digital formats, have demonstrated beneficial effects on SNK and, in some cases, on dietary intake among young female athletes (3, 7, 8, 18–28). However, evidence remains limited regarding short-term, feasible, and context-specific nutrition education interventions tailored to the needs of adolescent female team-sport athletes. Therefore, further research is required with well-designed and clearly described educational interventions, evaluated in accordance with guidelines such as GENIE (29), and that consider specific needs of female athletes.

Therefore, the aim of this study was to evaluate the effect of a short nutrition education intervention on SNK, dietary intake, and body composition in young athletes.

## 2 Materials and methods

### 2.1 Study design

This study was conducted as part of the Assessment of Training in Healthy Habits and Nutrition Education for Adolescent Athletes (ATHENEA) project, which received ethical approval from the Bioethics Committee of the University of Barcelona (initial protocol: IRB00003099, June 30, 2023; modified protocol: IRB00003099, October 14, 2025). A parallel-group cluster randomized controlled trial (RCT) in sports club settings was implemented to evaluate a three-week nutrition education intervention targeting adolescent female handball players in Catalonia, Spain. Clubs were assigned to either an intervention group (IG), which received the nutrition education program, or a control group (CG), which did not receive any intervention during the study period. Club-level allocation was necessary because the intervention was delivered in group sessions and individual randomization within clubs was not feasible due to the substantial risk of contamination arising from regular interaction and information sharing between players. The trial was prospectively registered at ClinicalTrials.gov (NCT07460102).

Prior to the development of the main study, a pilot project (30) was conducted between September 2023 and October 2024, which allowed for the refinement of the intervention protocol and the optimization of both the intervention procedures and the data collection strategies.

The study was conducted in accordance with the ethical principles outlined in the Declaration of Helsinki. All participants, as well as their parents and coaches, received a comprehensive description of the study, both orally and in writing. Participation was voluntary, and written informed consent was obtained from all participants and, in the case of minors under 16 years of age, from their parents or legal guardians, in accordance with Spanish legislation governing informed consent in minors. In all cases, participants’ consent took precedence, and parents or legal guardians were always informed. Additionally, the recommendations for the prevention of eating disorders on the practice of taking anthropometric measurements from children and adolescents were considered (31).

### 2.2 Randomization

Randomization was performed at the club level to prevent potential contamination between groups and ensure the internal validity of the study. Using the official registry of women’s handball clubs in Catalonia, each club was assigned a unique sequential numerical code, and selection was carried out randomly. Initial contact followed this numerical order and was established via email and telephone, culminating in an in-person meeting with club representatives and/or technical directors to explain the study objectives and formally invite participation. If a club declined the invitation, the next club on the sequential list was contacted until a total of four clubs agreed to participate. The four clubs were randomized to the study groups in a 1:1 ratio, allocating two clubs to the IG and two clubs to the CG. Random allocation was performed using a two-stage random selection and allocation procedure using Microsoft Excel.

While individual allocation was not concealed, outcome assessors and data analysts were blinded to group assignments. Participants, however, could not be blinded due to the inherent nature of the educational intervention.

### 2.3 Subjects

Participant recruitment was conducted between February and March 2025. Inclusion criteria were: (a) female athletes aged 12 to 19 years, (b) a minimum of 1 year of training experience at their club, and (c) training at least 3 days per week for approximately 1.5 h per day. Exclusion criteria comprised: (a) athletes who had not yet reached menarche, (b) a diagnosis of an eating disorder, (c) the presence of a chronic disease requiring a specific dietary plan, (d) pregnancy, and (e) lack of proficiency in Spanish. Inclusion and exclusion criteria were assessed through a self-reported questionnaire completed by the participants prior to enrollment.

### 2.4 Procedure

**Figure 1:**
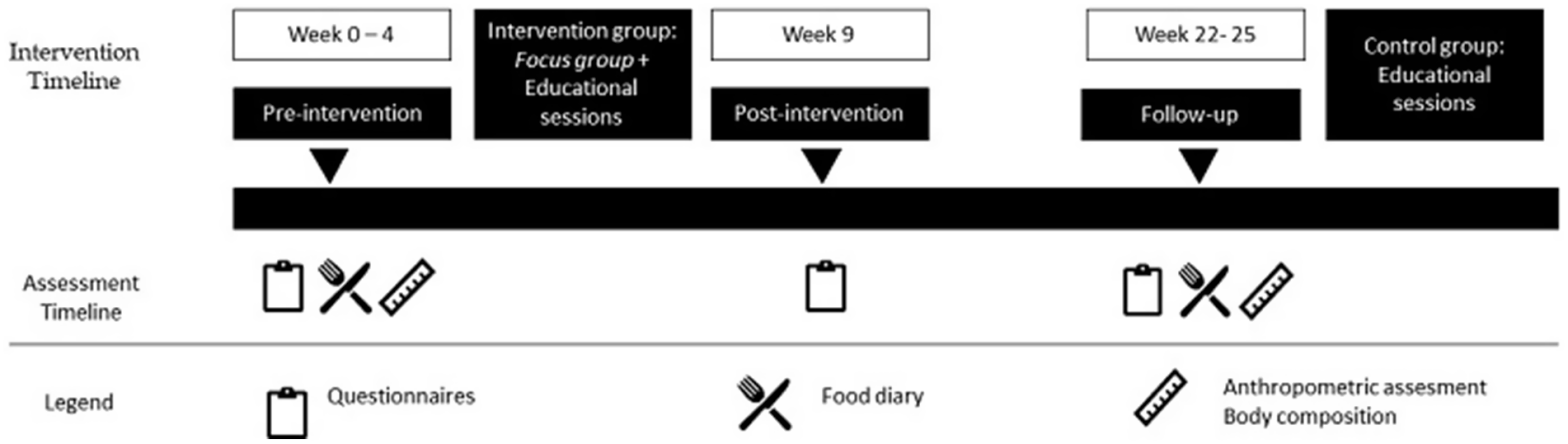
Study design and timeline.

The study was conducted from February 2024 to December 2025. The study design included four phases (Figure 1): pre-intervention, intervention, post-intervention, and a follow-up conducted 3 months after the completion of the intervention. The timing of the study phases was aligned with the competitive calendar of the participating sports clubs. The pre-intervention phase was conducted during the final period of the competitive season. The intervention phase took place across both the competitive and post-competitive periods, while the post-intervention assessments were performed during the post-competitive phase. The follow-up evaluation, conducted 3 months after the intervention, coincided with the beginning of the subsequent competitive season.

### 2.5 Intervention

During the initial contact with the athletes in the IG, a focus group session lasting 30 to 60 min was conducted to thoroughly explore their expectations, perceived barriers, and interests regarding nutrition. Guiding questions addressed topics such as dietary habits, challenges in maintaining healthy eating patterns, and preferred content for the intervention. The information obtained was used to inform the design and adaptation of the educational sessions.

The topics addressed in the intervention were determined from: (a) findings from the focus group, (b) results of the nutrition knowledge questionnaire administered during the pre-intervention phase, and (c) insights gained from the previously conducted pilot project. Additionally, the overall design of the intervention was reviewed using the Guide for Effective Nutrition Interventions and Education (GENIE) (29), to ensure alignment with evidence-based standards regarding program goals, instructional methods, content, and evaluation.

The nutrition education intervention consisted of three in-person group sessions delivered by a licensed dietitian-nutritionist, each lasting 30 min and delivered once per week. Its duration was established trying to maximize its viability in a real-world setting, outside the framework of a research study. Attendance was systematically recorded for each session. The content covered in these sessions is summarized in Table 1.

**Table 1:** Topics covered in the educational sessions.

Session	Topic
1	Introduction to health and the Mediterranean diet Macronutrients: Energy and carbohydrate requirements
2	Macronutrients: Protein and fat requirements Micronutrients: Vitamins and minerals
3	Hydration Supplementation and periodicity

Following the conclusion of the sessions, the visual materials used in each meeting along with a guide entitled “Nutrition for Young Athletes” were sent to the coaches via email, who distributed the guide to the players and their families. This document, prepared by a licensed dietitian in collaboration with final-year students of the Human Nutrition and Dietetics degree program at the University of Barcelona, included information and practical examples designed to optimize nutrition for sports performance.

The CG did not participate in any educational activities during the intervention phase. These participants were instructed to maintain their usual dietary habits and to avoid seeking additional nutritional information or counseling for the duration of the study. This group received the same nutrition education intervention upon completion of the study.

### 2.6 Data collection and measurements

#### 2.6.1 Questionnaires

All questionnaires were self-administered and completed online using forms designed with the REDCap (Research Electronic Data Capture) software platform installed in a server of the University of Barcelona. All questionnaires were completed under the supervision of the principal investigator to prevent discussion or exchange of information among respondents. Completion of the socio-demographic questionnaire, which collected baseline personal and demographic information, was required for inclusion in the study. This questionnaire collected baseline information including date of birth, competition category, educational level, years of training, employment status, parental educational level, main sources of nutrition information, previous individual nutritional counseling, and menstrual cycle history. The Eating Attitudes Test (EAT-26) (32), and the Body Shape Questionnaire (BSQ) (33) were administered only at baseline to assess risk of eating disorders and body image, respectively, as the intervention was not designed to target these outcomes. The EAT-26 was used as a screening tool and not for diagnostic purposes. SNK was evaluated using the Nutrition Knowledge Questionnaire for Young and Adult Athletes (NUKYA) (34) at baseline, post-intervention, and follow-up. Adherence to the Mediterranean diet (MD) was assessed with the KIDMED index (35), at baseline and follow-up.

#### 2.6.2 Assessment of dietary intake

The dietary intake assessment protocol was adapted from the pilot study (30), with minor adjustments to suit the design of the present RCT. Dietary intake was assessed using a three-day photographic food record, including two nonconsecutive weekdays and one weekend day corresponding to a match day. Data collection was conducted at baseline and during the follow-up phase using the mobile application Remind® (version 15.2) (36).

To ensure accurate reporting, participants attended a training session in which they received standardized instructions on the use of the application and the dietary recording procedure. Participants were instructed to photograph all foods and beverages consumed from a 45° angle using a fiducial marker (an object with known dimensions, such as a pen or cutlery) to facilitate portion size estimation. In addition to the photographs, participants were required to provide a brief description of the foods consumed and the time of intake.

The dietitians on the research teams reviewed the dietary records on a daily basis. Using the application Remind®, participants could be contacted in real time when photographs were incomplete or unclear, or when discrepancies between photographed and actual intake were suspected (e.g., partially consumed foods, foods removed before photography, or additional servings consumed after the photograph). Information obtained during this follow-up was incorporated into the dietary records and used to refine portion size estimations. As with all self-reported dietary assessment methods, some degree of misreporting cannot be fully excluded.

Dietary records were compiled and digitized using PCN Pro 1.0 software (37) by final-year students enrolled in the Human Nutrition and Dietetics degree program at the University of Barcelona, who were previously trained to ensure consistency and accuracy in data handling. Portion size estimation was supported by photographic guides based on foods commonly consumed in Spain (38).

Finally, average daily intakes of energy (kcal/day), macronutrients (g/day), and micronutrients (mg/day or μg/day) were calculated using Spanish food composition data (39). The micronutrients selected for analysis were those considered critical for female athletes: calcium, magnesium, iron, zinc, folate, vitamin C, vitamin D, vitamin E, vitamin B6, and vitamin B12. Final interpretation and analysis of the dietary data were performed by a registered dietitian.

#### 2.6.3 Anthropometric measurements and body composition

Body weight and height were measured using a mechanical column scale with an integrated stadiometer (model 711; maximum height 220 cm; accuracy 0.5 cm; Seca, Hamburg, Germany). Body composition, including fat mass (FM), muscle mass (MM), and fat-free mass (FFM), was assessed using a multifrequency bioelectrical impedance analysis device (BIA; Z-Metrix, Bioparhom, France), with six electrodes placed on the dominant side of the body at specific anatomical sites according to the manufacturer’s protocol.

All measurements were performed in the morning, with participants wearing underwear and following an overnight fast of at least 8 h. For body composition assessments, participants were instructed to avoid consuming coffee, tea, or other caffeine-containing beverages on the day of the evaluation, and to refrain from vigorous physical activity for at least 12 h beforehand. Prior to measurement, athletes were asked to empty their bladder, remove all metallic objects, and lie in a supine position with their limbs slightly separated from the body. Electrode placement followed the manufacturer’s specifications.

All assessments were carried out by a licensed dietitian at the respective sports facilities during both the baseline and follow-up phases.

### 2.7 Statistical analysis

The primary outcome was SNK. Secondary outcomes included adherence to the MD (KIDMED index), dietary intake variables, and anthropometric and body composition parameters. Outcome prioritization was informed by a single-arm pre–post pilot study (30). In the pilot, changes in KIDMED or anthropometric and body composition parameters were not statistically significant, whereas calcium intake was the only dietary variable that showed a trend toward improvement.

Sample size estimation for the primary outcome SNK assessed at baseline, post-intervention, and follow-up was conducted using the GLIMMPSE online tool (General Linear Mixed Model Power and Sample Size; University of Colorado Anschutz Medical Campus, Aurora, CO, United States), for a cluster-randomized, repeated-measures design. Power calculations targeted the group-by-time interaction for SNK across three assessments. For calcium intake measured at baseline and follow-up, additional calculations targeted the group-by-time interaction. Assumptions on the expected means, standard deviations, within-participant correlation across repeated measures, and intraclass correlation at the club level were based on pilot data and/or conservative estimates. The type I error rate was set at 0.05 and power at 80%. The minimum required total sample size was 14 participants for SNK and 74 participants for the secondary outcome calcium.

Statistical analyses were conducted according to a predefined plan adapted from our previously published pilot study (30). Data normality was assessed using the Shapiro–Wilk test, and homogeneity of variances was evaluated using Levene’s or Bartlett’s test, as appropriate. Between-group comparisons were performed using Student’s *t*-test for normally distributed variables or the Mann–Whitney *U* test for non-parametric variables.

Changes over time in SNK (NUKYA scores), adherence to the MD (KIDMED index), dietary intake variables, and anthropometric and body composition parameters were analyzed using linear mixed-effects models (LMMs) to account for the hierarchical structure of the data, with repeated observations nested within participants and participants clustered within clubs, consistent with the club-level randomization. This framework also allowed inclusion of incomplete observations under a likelihood-based approach. Given the small number of clubs (*n* = 4), club-level variance estimates were interpreted cautiously. Data missingness was explored descriptively by group and timepoint and further assessed in regression models including observed baseline covariates and previously observed data. No clear differences in missingness were observed by treatment group, and the presence of missing values at follow-up was associated with observed participant characteristics, supporting that the missing-data pattern was compatible with a missing-at-random assumption. However, this assumption cannot be formally verified from the observed data alone. *Post hoc* pairwise comparisons were adjusted using the Bonferroni correction. Effect sizes were calculated using Hedges’ *g* and interpreted as very small (<0.20), small (0.20–0.49), moderate (0.50–0.79), or large (≥0.80).

All statistical tests were two-tailed, with a confidence level set at 95%. Statistical significance was defined as *p* < 0.05. Analyses were performed using STATA statistical software (version 16.1; StataCorp, College Station, TX, United States).

## 3 Results

A total of 229 players from the selected clubs were invited to participate in the study, of whom 101 provided informed consent. Four players were excluded because they had not yet experienced menarche. Participants were categorized according to the competitive levels defined by the Royal Spanish Handball Federation: alevina (Under 12; 11–12 years), infantil (Under 14; 13–14 years), cadete (Under 16; 15–16 years), juvenil (Under 18; 17–18 years), and senior (>18 years).

**Figure 2:**
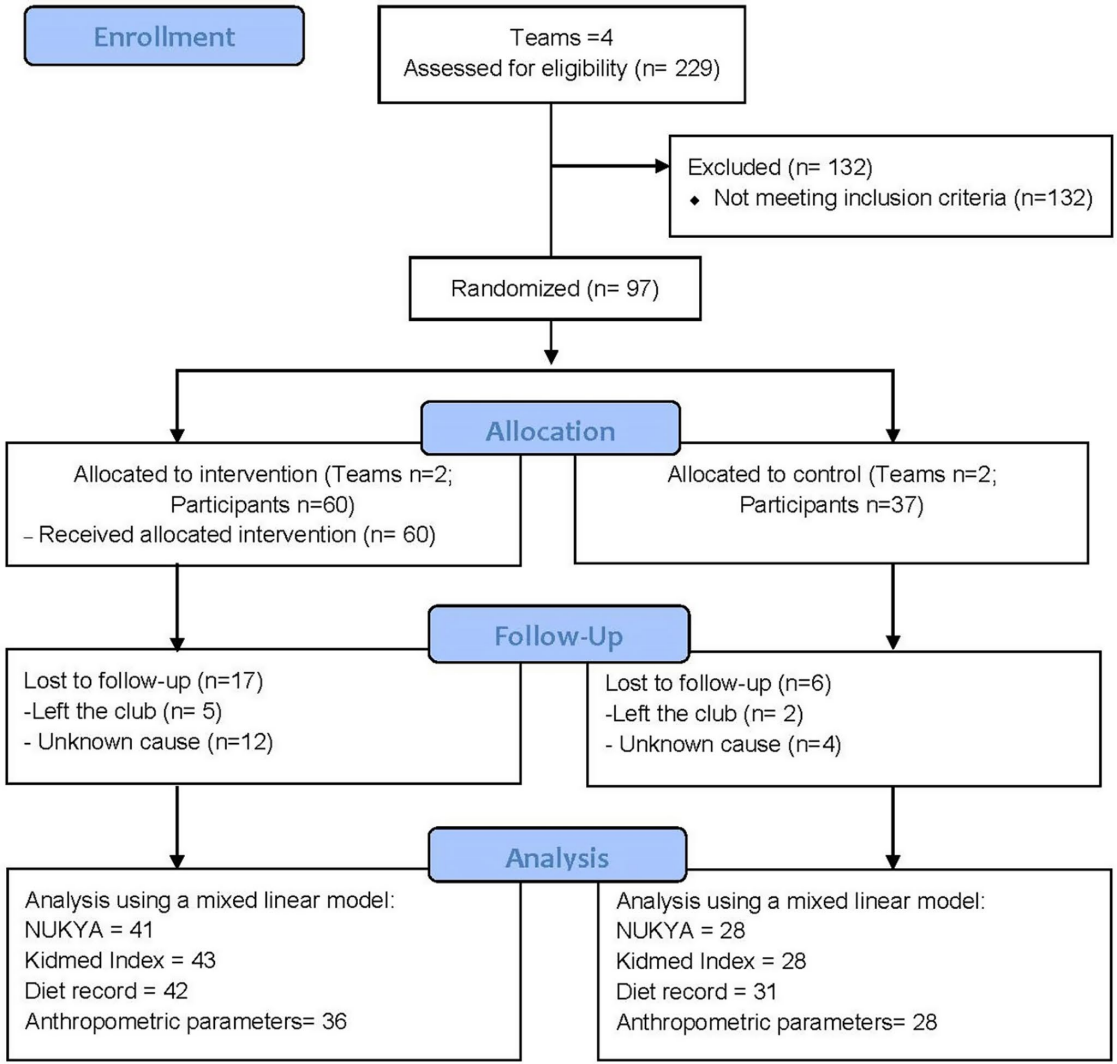
Consort flow diagram.

During the follow-up phase, 17 participants withdrew from the study in the IG and 6 in the CG. No statistically significant differences were detected between missing participants and completers in the baseline data. The flow of participants throughout the study, is illustrated in the CONSORT flow diagram (Figure 2).

Overall, 97 participants were included in the study, with 60 assigned to the IG and 37 to the CG. Participants’ ages ranged from 12 to 19 years, with a mean of 15.9 ± 1.2 years (Table 2). No statistically significant differences in age were observed between groups. Most participants in both groups belonged to the cadet category and had more than 5 years of training experience. Participants trained three times per week for 1.5 h per session, in addition to one competitive match during the weekend, with no differences between the intervention and control groups.

**Table 2:** Demographic characteristics of participants at baseline (*N* = 97).

Characteristics	Intervention group (IG) *n* = 60	Control group (CG) *n* = 37
Age (years; mean and s.d.)	16.0 (1.0)	15.6 (1.5)
Competition category (*n*)		
Alevina (Under 12; 11–12 years)	0	1
Infantil (Under 14; 13–14 years)	0	5
Cadete (Under 16;15–16 years)	38	16
Juvenil (Under 18; 17–18 years)	22	14
Senior (>18 years)	0	1
Team rol (*n*)		
Player	58	37
Goalkeeper	2	0
Training period (*n*)		
1–3 years	9	4
3–5 years	12	8
>5 years	39	25
Level of education (*n*)		
Basic	38	22
Intermediate	22	14
University	0	1
Currently employed (*n*)		
Yes	2	1
No	58	36
Mother’s highest educational level (*n*)		
Without studies or only compulsory education	4	3
Secondary education	18	7
University education	38	27
Father’s highest educational level (*n*)		
Without studies or only compulsory education	10	5
Secondary education	20	12
University education	30	20
Sources of nutritional information used (*n*)		
Family	44	31
Coach	2	0
Dietitian-Nutritionist	8	6
Attendance at sessions (*n*, %)		
Session 1	39 (65%)	–
Session 2	42 (70%)	–
Session 3	36 (60%)	–
Completion of questionnaires (*n*)		
EAT-26	60	37
BSQ	60	37
NUKYA pre-intervention	58	35
NUKYA post-intervention	56	35
NUKYA follow-up	41	28
KIDMED Index pre-intervention	59	36
KIDMED Index follow-up	43	28
Dietary record pre-intervention	57	35
Dietary record follow-up	42	31
Anthropometric parameters pre-intervention	59	35
Anthropometric parameters follow-up	36	28

EAT-26, eating attitudes test; BSQ, body shape questionnaire; NUKYA, sports nutrition knowledge questionnaire for young and adult athletes.

Regarding the assessment of eating behavior, the mean EAT-26 score was 7.9 ± 10.5 in the IG and 6.6 ± 7.5 in the CG. A total of 8.3% (*n* = 5) of the IG and 5.4% (*n* = 2) of the CG scored above 20 points, indicating a high risk of eating disorders. These participants were not excluded from the study, as no clinical diagnosis of an eating disorder was present, in accordance with the exclusion criteria. Cases were reported to the respective clubs, and subsequent clinical evaluation did not confirm the presence of an eating disorder. For body image, assessed using the BSQ, mean scores were 39.8 ± 15.5 in the IG and 36.6 ± 15.9 in the CG. High levels of body dissatisfaction were observed in 38.3% (*n* = 23) of the IG and 27.0% (*n* = 10) of the CG. No statistically significant differences were found between groups at baseline for either questionnaire.

### 3.1 Sports nutritional knowledge

Changes in SNK scores over time for both groups are presented in Table 3. At baseline (pre-intervention), no significant differences were observed between the IG and the CG in total questionnaire scores.

**Table 3:** Sport nutrition knowledge (SNK) total score, domain scores and change scores at each time period.

	Intervention group (IG, *n* = 60)			Control group (CG, *n* = 37)			*p*-value (group × time interaction)
	Pre M (SD)	Post M (SD)	Follow-up M (SD)	Pre M (SD)	Post M (SD)	Follow-up M (SD)	Pre vs. Follow-up
Total and section scores	60			37			
Total score	*n* = 58 24.2 (16.1)	*n* = 56 43.6 (20.5)	*n* = 41 41.8 (19.3)	*n* = 35 25.3 (21.0)	*n* = 35 29.6 (17.9)	*n* = 28 29.5 (14.4)	0.001*
Sections							
Macronutrients	15.0 (10.8)	22.5 (12.2)	22.0 (12.5)	15.6 (13.0)	19.0 (11.3)	17.9 (10.0)	0.091
Micronutrients	9.7 (6.9)	12.9 (7.4)	13.9 (6.9)	7.6 (8.1)	10.1 (6.7)	10.4 (5.8)	0.294
Hydration	−1.1 (3.4)	6.1 (5.4)	3.3 (3.3)	0.6 (3.5)	−0.2 (3.1)	0 (4.0)	<0.001*
Periodization	0.6 (2.9)	2.1 (2.8)	2.5 (2.4)	1.5 (2.9)	0.7 (2.7)	1.3 (2.7)	0.015*

M, mean; SD, standard deviation; *significantly different at *p* < 0.05.

In the IG, scores on the NUKYA significantly increased from baseline to post-intervention by 19.7 points (95% CI: 15.1 to 24.2; *p* < 0.001, Hedges’ *g* = 1.05) and from baseline to follow-up by 16.8 points (95% CI: 11.7 to 21.8; p < 0.001, Hedges’ *g* = 1.00), whereas no significant changes were observed in the CG. The intraclass correlation coefficient (ICC) indicated that approximately 56% of the total variance in NUKYA scores was attributable to differences between individuals, whereas variance between clubs was negligible (ICC ≈ 0). No adjustments were made for potential confounders, as the IG and CG were homogeneous in terms of age, educational level, and maternal education. The group × time interaction was significant (post-intervention: *β* = 15.6, *p* < 0.001; follow-up: *β* = 13.2, *p* = 0.001), indicating a differential effect of the intervention over time.

Furthermore, the IG exhibited significant improvements across all four questionnaire domains from baseline to post-intervention and from baseline to follow-up (*p* < 0.001). However, the group × time interaction reached statistical significance only for the hydration and periodization domains (*p* < 0.005), indicating that the intervention had a differential impact over time specifically for these areas.

### 3.2 Mediterranean diet score adherence

At baseline, 59 participants in the IG and 36 in the CG completed the KIDMED questionnaire. The mean baseline score was 5.6 ± 2.4 in the IG and 5.7 ± 1.8 in the CG, with no significant difference between groups. Optimal adherence to the MD was observed in 22.0% (*n* = 13) of IG participants and 22.2% (*n* = 8) of CG participants. The majority of participants in both groups required improvements to better align their diet with Mediterranean patterns (62.7% in IG and 66.7% in CG), while 15.3% (*n* = 9) and 11.1% (*n* = 4), respectively, exhibited very low adherence.

Analysis of specific dietary behaviors at baseline revealed that only 33.7% of participants consumed a second piece of fruit daily, 32.6% included fresh or cooked vegetables more than once per day, and 34.7% consumed the recommended two servings of dairy products (yogurt or 40 g cheese) per day. In contrast, 33.7% consumed industrial pastries, cookies, or cakes at breakfast, and 14.7% reported consuming candies or sweets multiple times per day.

During the follow-up, 43 participants in the IG and 27 in the CG completed the questionnaire. After the intervention, KIDMED scores tended to increase in both groups; however, this increase was statistically significant only in the IG (*Δ* = 0.8; IC 95% = 0.26 a 1.29; *p* = 0.003). The group × time interaction was not statistically significant (*β* = 0.48; *p* = 0.253), indicating that changes over time did not differ significantly between the IG and the CG.

### 3.3 Dietary intake assessment

At baseline, 57 participants in the IG and 35 in the CG completed the 3-day dietary record. No statistically significant differences were observed between groups for any dietary variable at this initial phase. Tables 4, 5 present changes in energy, macro-, and micronutrient intake over the course of the study for both groups. At follow-up, the analyses did not reveal any dietary variable with a significant group × time interaction, indicating that the intervention did not result in differential changes in dietary intake between groups over time. However, a significant main effect of time was observed for saturated fat intake, which decreased significantly in both groups. Specifically, saturated fat intake was reduced in the IG (−2.7 *g*; *p* = 0.011) as well as in the CG (−3.4 *g*; *p* = 0.007). In both groups, most of this decrease (65% in the IG and 55% in the CG) was due to a reduction in SFA intake coming from dairy products, sauces, oils, sugary products (including chocolate), and beverages (including juices with added milk). However, a significant portion of the reduction in SFA intake (35% in the IG and 45% in the CG) showed a differential distribution between groups: in the IG, this reduction was due to lower intake of SFA from cereal products (including pastries) and eggs, whereas in the CG it was mainly explained by a decrease in SFA from meat and nuts. However, it should be noted that analyses of food group intake (expressed as grams per day) showed no significant main effects of time and no significant group × time interactions, indicating that food group consumption remained stable in both groups throughout the study.

**Table 4:** Energy and macronutrient intake at pre-intervention and in the follow-up period.

Energy and main dietary components	Intervention group (IG)		Control group (CG)		*p*-value (group × time interaction)
	Pre M (SD)	Follow-up M (SD)	Pre M (SD)	Follow-up M (SD)	
	*n* = 57	*n* = 42	*n* = 35	*n* = 31	
Energy (kcal)	1780.0 (331.1)	1731.0 (320.2)	1762.7 (435.3)	1657.9 (309.6)	0.767
CHO (g/d)	159.0 (39.6)	161.2 (38.0)	153.6 (42.0)	152.5 (38.7)	0.933
CHO (% kcal)	35.7 (5.6)	37.0 (5.1)	35.0 (5.6)	36.6 (5.5)	
Fiber (g/d)	14.0 (5.0)	13.4 (3.6)	13.9 (3.7)	13.2 (4.1)	0.895
Protein (g/d)	86.4 (18.4)	85.1 (16.5)	86.3 (21.7)	84.0 (18.3)	0.936
Protein (% kcal)	19.5 (3.0)	19.8 (2.4)	19.8 (2.9)	20.3 (2.9)	
Lipids (g/d)	88.5 (20.6)	82.8 (17.7)	89.1 (27.0)	79.0 (17.6)	0.527
Lipids (% kcal)	44.7 (5.0)	43.1 (5.1)	45.2 (5.7)	43.0 (5.3)	
SFA (g/d)	27.2 (7.5)	24.5 (6.0)	27.1 (9.1)	23.2 (6.1)	0.673
SFA (% kcal)	13.7 (2.3)	12.7 (1.8)	13.7 (2.5)	12.6 (2.4)	
MUFA (g/d)	38.4 (11.1)	36.6 (8.4)	39.5 (13.1)	34.4 (8.4)	0.195
MUFA (% kcal)	19.4 (3.7)	19.1 (2.9)	20.0 (3.6)	18.7 (3.0)	
PUFA (g/d)	14.7 (6.1)	13.3 (3.6)	14.5 (6.2)	13.3 (3.5)	0.820
PUFA (% kcal)	7.4 (2.7)	7.0 (2.0)	7.4 (2.5)	7.2 (1.7)	
Cholesterol (mg/d)	360.4 (132.4)	358.1 (157.3)	360.6 (174.3)	351.9 (152.4)	0.940

M, mean; SD, standard deviation; CHO, carbohydrates; SFA, saturated fatty acids; MUFA, monounsaturated fatty acids; PUFA, polyunsaturated fatty acids.

**Table 5:** Micronutrient intake pre-intervention and during the follow-up period.

Micronutrients	Intervention group (IG)		Control group (CG)		*p*-value (group × time interaction)
	Pre M (SD)	Follow-up M (SD)	Pre M (SD)	Follow-up M (SD)	
	*n* = 57	*n* = 42	*n* = 35	*n* = 31	
Calcium (mg)	707.9 (221.5)	711.1 (200.2)	648.0 (211.2)	693.9 (171.6)	0.434
Magnesium (mg)	246.2 (59.6)	243.6 (56.0)	245.7 (52.9)	236.4 (50.8)	0.636
Iron (mg)	10.9 (2.8)	10.2 (2.2)	10.7 (3.0)	9.7 (2.6)	0.688
Zinc (mg)	8.9 (2.2)	8.6 (2.0)	8.9 (2.3)	8.6 (2.2)	0.848
Folates (μg)	200.2 (70.5)	180.7 (49.1)	206.3 (76.4)	168.9 (49.6)	0.220
Vitamin C (mg)	69.0 (43.9)	59.5 (27.9)	74.4 (47.9)	54.5 (33.6)	0.194
Vitamin D (μg)	2.3 (1.8)	2.2 (1.7)	2.5 (2.2)	3.0 (2.5)	0.360
Vitamin E (mg *)	10.6 (4.4)	9.3 (2.7)	10.2 (5.1)	9.2 (3.1)	0.802
Vitamin B6 (mg)	1.7 (0.5)	1.7 (0.4)	1.7 (0.5)	1.6 (0.4)	0.995
Vitamin B12 (μg)	4.3 (1.7)	4.4 (2.7)	4.9 (2.5)	4.5 (1.8)	0.485

M, mean; SD, standard deviation; *Alfatocopherol equivalents.

### 3.4 Anthropometric measurements and body composition

A total of 59 participants in the IG and 35 in the CG underwent anthropometric and body composition assessments at baseline. No significant differences were observed between groups in any of the variables evaluated at this time. Table 6 presents the body composition measurements for both groups at baseline and at the follow-up phase. No variables showed significant group × time interactions across the study period.

**Table 6:** Body composition parameters pre-intervention and in the follow-up period.

	Intervention group (IG)		Control group (CG)		*p*-value (group × time interaction)
	Pre M (SD)	Follow-up M (SD)	Pre M (SD)	Follow-up M (SD)	
Body composition parameters	*n* = 59	*n* = 36	*n* = 35	*n* = 28	
Weight (kg)	59.0 (6.6)	60.7 (6.8)	58.1 (8.3)	59.6 (8.3)	0.136
Fat mass (%)	29.6 (6.2)	29.9 (5.2)	28.9 (5.7)	29.5 (5.1)	0.726
Fat free mass (%)	70.4 (6.2)	70.1 (5.2)	71.1 (5.7)	70.5 (5.1)	0.662
Muscle mass (%)	34.4 (3.9)	35.7 (3.9)	35.7 (3.4)	35.8 (2.4)	0.105

M, mean; SD, standard deviation.

## 4 Discussion

The present RCT evaluated the effectiveness of a nutrition education intervention on SNK, dietary intake, and body composition in female handball players compared with a non-intervention CG. The intervention produced a significant increase in SNK in the IG with a large effect size, although changes in dietary intake and body composition were not observed, a pattern commonly reported in short-term interventions among adolescents. Importantly, this study was conducted using a brief, context-specific intervention tailored to the needs of the athletes, which enhances its practical applicability in real-world sports settings.

### 4.1 Sports nutritional knowledge

At baseline, both groups showed insufficient SNK levels, consistent with previous studies using the same questionnaire (17, 30). In the pilot phase of this project, a mean score of 21.1 ± 16.1 was reported (30), while a similar score (22.8 ± 13.3) was found in non-athlete Spanish women (17), indicating that low SNK is prevalent among adolescent females regardless of athletic status. This finding is relevant given the potential of organized sports environments as platforms for nutrition education during a critical developmental stage.

Low baseline SNK may be partly explained by the sources of nutrition information. Participants mainly relied on family members (77%), while only 14% consulted a registered dietitian-nutritionist, consistent with previous research (17, 28, 40, 41). The family may provide early guidance but, when nutritional knowledge is limited, may also perpetuate misconceptions. In this context, structured nutrition education appears to be an effective strategy for improving SNK. In the present study, SNK increased by 19.7 points in the IG (Hedges’ *g* = 1.05), representing a greater magnitude of change than that reported in similar recent interventions (8, 42). In comparison, present improvements were achieved through a relatively brief and low-intensity program, suggesting that well-designed educational interventions can be efficient, effective, and feasible in youth sports settings.

The consistent improvements in SNK observed in both the pilot study (30) and the present RCT align with evidence synthesized in a recent systematic review, which demonstrated that structured nutrition education interventions lead to significant improvements in SNK among young female athletes across a wide range of sports disciplines (43). These results indicate that nutrition education can play an important supportive role in the athletic development process by enabling young athletes to make more informed dietary decisions related to health and sport performance.

Despite the positive effects reported across studies, the existing literature highlights considerable heterogeneity in the design and implementation of nutrition education interventions. Variability in delivery modality, session frequency, duration, and the professional background of educators limits the identification of the specific components driving intervention success and reduces comparability across studies. This issue was highlighted in the aforementioned systematic review (43) and further supported by DeJong et al. (44), who reported substantial variability in interventions targeting SNK and low energy availability in athletes over 18 years of age. Notably, the most favorable outcomes were associated with face-to-face interventions consisting of at least two sessions and delivered by qualified professionals with expertise in sports nutrition. Similarly, Gao et al. (45) compared face-to-face and online nutrition education interventions in adolescent football players and found that both modalities improved SNK; however, the face-to-face intervention comprising 12 group sessions of 30 min delivered by a registered dietitian produced significantly greater and more consistent learning effects. It should be noted that in-person and prolonged interventions are less feasible due to the financial limitations of the clubs and greater interference with team activities.

### 4.2 Mediterranean diet score adherence

At baseline, 78.0% of players in the IG and 77.8% in the CG required improvement in their adherence to the MD, reflecting moderate or low adherence levels. These findings are consistent with previous reports in handball and beach handball players, in which between 82 and 85% required improvements in their diet (30, 46), and with a recent systematic review by Alfaro-González et al. (47) reported a decline in MD adherence in the Spanish population aged 2–24 years between 2011–2014 and 2019–2023, with high adherence decreasing from 41.4 to 24.3% and low adherence increasing from 9.1 to 13.8%. In this context, adolescents and young adults (12–24 years) showed relatively low adherence, with only 36% achieving high adherence. This decline in MD adherence has been associated with a complex interaction of factors, including socio-economic conditions, lifestyle behaviors, and environmental influences. In younger populations, eating habits are shaped by family and social contexts, as well as by broader societal changes such as urbanization, technological development, and the increased availability and promotion of ultra-processed foods, which have contributed to a shift away from traditional dietary patterns (48).

The analysis of specific dietary behaviors observed in this study is consistent with these patterns and helps to explain the moderate-to-low adherence to the MD in this population. These results suggest intakes below recommended levels for fruits, vegetables, and dairy products. Moreover, these findings highlight a frequent intake of sugar-rich foods. These patterns are consistent with prior studies in female athletes (30, 46, 49).

Although the educational intervention in the IG resulted in a significant improvement in SNK, no significant changes were observed in KIDMED scores, indicating that adherence to the MD did not improve. A similar situation was reported by Vicente et al. (50).

From a theoretical perspective, higher levels of SNK would be expected to promote better adherence to the MD. This assumption is supported by Barcın et al. (51), who reported a weak but positive correlation between SNK and MD adherence in a sample of 400 physically active individuals, suggesting that individuals with greater nutrition knowledge are more likely to follow the MD. Likewise, the systematic review by Janiczak et al. (10) reported weak-to-moderate positive associations between SNK and healthy dietary behaviors. All this evidence indicates that translating improved nutrition knowledge into sustained dietary behavior change remains challenging.

### 4.3 Dietary intake assessment

In the present community-based RCT, the nutrition education intervention did not elicit significant changes in energy, macronutrient, micronutrient, or food group intake compared with the CG. Linear mixed-effects analyses showed no dietary changes over the study period. These findings are consistent with our pilot study (30), in which limited improvements in actual dietary intake were observed.

At pre-intervention, mean energy intake in both groups was approximately 1,770 kcal/day (30 kcal/kg/day), comparable to values reported in other studies with female athletes from different sports disciplines (7, 8, 23, 24, 52, 53).

Carbohydrate intake was also suboptimal at both assessment points, contributing approximately 35% of total energy intake and averaging 2.7 g/kg/day, values below current recommendations for athletes with moderate-to-high training loads (6). These findings highlight the need for ongoing nutritional strategies to improve carbohydrate intake, given its role in athletic performance and glycogen replenishment. Dietary fiber intake (14.1 ± 4.5 g/day) was also well below current recommendations (25–35 g/day) (54) in line with findings from previous studies (7, 27). Protein intake remained within recommended ranges throughout the study (1.2–2.0 g/kg/day) (6) with mean intakes consistent with previous research on female athletes. Total fat intake exceeded recommendations throughout the study (45% of energy), and although saturated fat intake decreased significantly over time in both groups, this change seems to be attributed more to seasonality of the diet (55). The above described pattern (excess fat combined with low carbohydrate and fiber intake) are consistent with previous reports in female athletes and could negatively impact both performance and long-term health. Regarding micronutrient intake, no significant intervention-related improvements were observed for nutrients commonly identified as critical for female athletes, including iron, calcium, and vitamin D.

Overall, these findings indicate that improvements in SNK do not automatically translate into changes in dietary intake during a relatively short intervention period. Tektunali et al. (8) implemented a similar group-based educational program comprising six weekly 60-min sessions and observed significant improvements in dietary intake among young female athletes. The contrast in outcomes between studies may be attributable to differences in total exposure time, intensity of content delivery, opportunities for practical skill reinforcement, and contextual factors such as participant engagement and environmental support.

### 4.4 Anthropometric measurements and body composition

Our findings indicate that participants’ anthropometric and body composition measurements did not show significant changes following the intervention, consistent with observations from the pilot study (30) and other research in young female athletes (22, 27, 52). This stability may reflect participants’ favorable baseline body composition, indicative of the fitness required to meet training demands and competition. Additionally, the short duration of the present intervention (three group sessions) and ongoing adolescent growth likely limited observable changes.

Three previous studies in female athletes reported significant improvements in body composition following nutrition education interventions (7, 23, 56), with program durations ranging from 4 to 8 months. These findings suggest that intervention length and intensity are critical for translating nutrition knowledge into measurable physiological outcomes. Notably, these studies also reported improvements in dietary intake, supporting the notion that behavioral changes in nutrition are necessary to elicit changes in body composition. In our study, the absence of dietary intake improvements likely contributed to the lack of compositional changes.

### 4.5 Strengths and limitations

One of the main strengths of this study is its robust experimental design, which included an IG and a CG, with randomization of sports clubs, helping to minimize bias and enhance the internal validity of the results. Secondly, the nutrition education program was previously developed and tested in a pilot study by the same research group in a population with similar characteristics, ensuring that the intervention was evidence-based and appropriate for the target group.

Furthermore, the educational sessions were designed and delivered by a registered dietitian with experience in sports nutrition and were conducted in person, facilitating direct interaction and comprehension of the content. In addition, the session topics were tailored based on the results of an initial nutrition knowledge questionnaire and the specific interests of the participants, identified through a prior focus group. This strategy allowed the content to be adapted to the real needs and preferences of the female athletes, ensuring the relevance and personalization of the intervention.

Validated tools in a Spanish population were also used to assess nutrition knowledge and adherence to the MD, ensuring the reliability and accuracy of the measurements. Additionally, an average attendance rate above 60% ensured sufficient exposure to the intervention, supporting the effectiveness of the delivered content.

Finally, a key strength of this study lies in its real-world applicability, as the intervention was designed to be integrated into routine sports club settings. By aligning with the time and resource constraints typical of these environments, the program offers a feasible approach that can be realistically implemented and replicated in similar populations.

Despite these strengths, the study has some limitations. First, parents were not included in the educational sessions. This was mainly due to organizational constraints, as it was not feasible to schedule sessions that accommodated the wide range of parents’ working hours and other commitments. Although parents did not participate directly, informational materials were provided to ensure access to the educational content and to support the reinforcement of key messages outside the sessions. Second, dietary records were self-reported, which may have introduced recall bias or underreporting of intake, limiting the precision of dietary assessments. Additionally, although the sample size estimation indicated that a minimum of 74 participants was required to adequately power the analysis of dietary variables, participant attrition resulted in a final sample of 73 individuals. This slight shortfall may have reduced the statistical power to detect group-by-time differences in dietary outcomes. Lastly, there were participant losses during the study, mainly due to changes of sports clubs, discontinuation of sports activity for personal reasons, or loss of interest after receiving the intervention, which could affect the sample size and the generalizability of the results.

## 5 Conclusion

This RCT demonstrates that a brief, three-week nutrition education intervention is effective in significantly improving SNK among adolescent female handball players. However, these knowledge gains did not translate into measurable changes in dietary intake or body composition during the follow-up period, indicating that information acquisition alone may be insufficient to induce short-term behavioral or physiological changes in this population.

Baseline findings revealed the coexistence of important gaps in SNK and suboptimal dietary patterns, reinforcing the need for structured nutrition education programs led by qualified professionals within youth sports settings. Such programs are particularly relevant for adolescent athletes, who face increased nutritional demands related to growth, training, and competition, and who may lack access to evidence-based guidance.

Future interventions should incorporate longer follow-up periods, greater educational intensity, and the integration of behavioral, motivational, and environmental components to facilitate the translation of knowledge into practice. In addition, the implementation of evidence-based nutrition education programs delivered by qualified professionals is essential to ensure the quality and reliability of the intervention content. Establishing clearer frameworks that define core components, such as essential content, minimum exposure, and delivery format, may further enhance the effectiveness, comparability, and translational potential of future interventions. Strategies such as individualized counseling, practical skill development, and engagement with coaches and family members may be particularly important to promote sustainable improvements in energy and nutrient intake. Overall, these findings highlight that while nutrition education is a necessary foundation, comprehensive and multifaceted approaches are required to optimize dietary behaviors, health, and performance in young female athletes.

## Data Availability

The raw data supporting the conclusions of this article will be made available by the authors, without undue reservation.
